# Fall risk as a function of time after admission to sub-acute geriatric hospital units

**DOI:** 10.1186/s12877-016-0346-7

**Published:** 2016-10-07

**Authors:** Kilian Rapp, Johannes Ravindren, Clemens Becker, Ulrich Lindemann, Andrea Jaensch, Jochen Klenk

**Affiliations:** 1 Department of Clinical Gerontology, Robert-Bosch-Hospital, Auerbachstr. 110, 70376 Stuttgart, Germany; 2 Institute of Epidemiology and Medical Biometry Ulm, Ulm University, Ulm, Germany; 3 Department of Neurology, Klinikum Stuttgart, Germany

**Keywords:** Accidental Falls, Rehabilitation Centers, Confusion, Femoral Fractures, Stroke

## Abstract

**Background:**

There is evidence about time-dependent fracture rates in different settings and situations. Lacking are data about underlying time-dependent fall risk patterns. The objective of the study was to analyse fall rates as a function of time after admission to sub-acute hospital units and to evaluate the time-dependent impact of clinical factors at baseline on fall risk.

**Methods:**

This retrospective cohort study used data of 5,255 patients admitted to sub-acute units in a geriatric rehabilitation clinic in Germany between 2010 and 2014. Falls, personal characteristics and functional status at admission were extracted from the hospital information system. The rehabilitation stay was divided in 3-day time-intervals. The fall rate was calculated for each time-interval in all patients combined and in subgroups of patients. To analyse the influence of covariates on fall risk over time multivariate negative binomial regression models were applied for each of 5 time-intervals.

**Results:**

The overall fall rate was 10.2 falls/1,000 person-days with highest fall risks during the first week and decreasing risks within the following weeks. A particularly pronounced risk pattern with high fall risks during the first days and decreasing risks thereafter was observed in men, disoriented people, and people with a low functional status or impaired cognition. In disoriented patients, for example, the fall rate decreased from 24.6 falls/1,000 person-days in day 2–4 to about 13 falls/1,000 person-days 2 weeks later. The incidence rate ratio of baseline characteristics changed also over time.

**Conclusions:**

Fall risk differs considerably over time during sub-acute hospitalisation. The strongest association between time and fall risk was observed in functionally limited patients with high risks during the first days after admission and declining risks thereafter. This should be considered in the planning and application of fall prevention measures.

**Electronic supplementary material:**

The online version of this article (doi:10.1186/s12877-016-0346-7) contains supplementary material, which is available to authorized users.

## Background

It seems obvious that individual fall risks could change within short time periods. Therefore, it is remarkable that there are no studies which analysed fall rates as a function of time in a systematic way. In fracture epidemiology there is some evidence about time-dependent risks. Two previous studies, for example, demonstrated that the first time after admission to a nursing home is a high-risk situation for fragility fractures [1, 2]. In these studies the fracture risk after admission was highest during the first weeks and declined thereafter. Potential causes of the observed pattern may have been the new environment which is a challenge to many of the new and often cognitively impaired residents. They are not used to the bedroom, the way to the toilet or may have difficulties finding the light switch. These aspects may have been responsible for an increased risk of falling. Another study in old community-dwelling people found that the first weeks after discharge from hospital were associated with an increased risk for femoral fractures [3]. A morbidity-related weakness with a deterioration of gait and balance, and a still (sub-acute) delirium may be further reasons for a transient increased risk of falling which could explain the observed time-dependent pattern. All the above mentioned intrinsic and extrinsic reasons for falls could be also present when people are admitted to hospital or transferred to a rehabilitation clinic. Therefore, time-dependent fall risks may be also found in hospitalised patients. This is of specific interest in geriatric patients transferred to a rehabilitation clinic since their fall rates have been reported to be particular high [4, 5]. In addition, rehabilitation may have contrary impacts on fall rates. On the one hand, exercise improves strength, balance and gait, on the other hand increasing physical activity increases the time at risk. Finally, specific subgroups of patients being in rehabilitation may have completely different time-dependent patterns of fall risk. This could be of relevance for the initiation of subgroup-specific preventive measures.

Many studies reported fall rates in hospitalised patients [6–11]. A few of these studies suggested even higher fall rates during the first days of hospitalisation [8–10]. These studies, however, did not analyse this topic systematically and did usually not consider changing person-days at risk due to discharge, transferral or death. There are no studies so far which analysed fall rates or risk factors for falls as a function of time after admission to an acute or sub-acute hospital in a systematic way.

In our study, we analysed a) fall rates as a function of time after admission to a geriatric rehabilitation clinic and b) the time-dependent impact of different clinical baseline factors on fall risk in more than 5,200 hospitalised patients.

## Methods

### Setting and study population

The analyses were performed with an anonymized dataset which included all patients admitted to one geriatric rehabilitation clinic in south-west Germany between 1.01.2010 and 31.12.2014. Geriatric rehabilitation is usually preceded by a stay in an acute care hospital. Patients with femoral fracture, for example, are usually transferred to the geriatric rehabilitation clinic after 10 − 14 days of acute care. Frequent reasons for geriatric rehabilitation are fragility fractures such as hip fracture, neurological diseases such as stroke, lower limb amputation, or cardiovascular diseases such as myocardial infarction or congestive heart failure. The length of stay is usually 3 weeks but can be extended by 1, 2 or in rare cases even more weeks.

### Baseline characteristics

All variables were retrospectively extracted from the electronic hospital information system. The Barthel-Index (BI) is a widely used, standardized tool for measuring functional status. Patients are scored in ten different activities of daily living (ADL) upon their independence of performance. The total score ranges from 0 (complete dependence) to 100 (complete independence) [12]. DemTect is a screening tool to identify patients with mild cognitive impairment and dementia in the early stages of the disease. It includes 5 tasks and its score ranges from 0 to 18 points [13].

The degree of orientation and the Barthel-Index were assessed and recorded by a nurse at the admission day. Cognition (DemTect) was assessed by an occupational therapist during the first week of stay. For the analyses the variables were dichotomised (fully oriented vs. not fully oriented; strong cognitive impairment (DemTect 0–8 points) vs. slight or no cognitive impairment (DemTect ≥9 points); low functional status (BI <60) vs. better functional status (BI ≥60)). The cut-off point of the Barthel-Index was chosen according to the median value within the study population.

We did not include other measures of single activities of daily living like transfer scales or gait speed since they were already represented by the Barthel-Index or could not be performed by a large percentage of patients at admission due to substantial functional impairments.

### Falls

Each fall at the geriatric rehabilitation clinic has to be electronically recorded by nurses and confirmed by a physician. Information about the exact date and time of the fall is available. For the analyses these recorded falls were extracted from the electronic hospital information system.

### Statistics

If a patient had 2 or more rehabilitation stays within the study period, each stay was handled independently. All falls of a patient were included in the dataset. Time at fall risk at the day of admission and the day of discharge is clearly less than 24 h and varies considerably between different patients due to organisational reasons. Therefore, the day of admission and the day of discharge were not included in the analyses. Only the actual time at fall risk was considered for the analyses. If a patient, for example, had to be transferred to an acute ward at day 10, only the days 2–9 were used for the analyses. To analyse fall risk during rehabilitation as a function of time after admission time-intervals were defined. For the descriptive analyses 3-day-intervals were chosen as a trade-off between temporal resolution and fall numbers. In addition, a subgroup analysis was performed which was restricted to patients who got an extension of their stay beyond the usually granted rehabilitation period of 3 weeks. The fall rate was calculated by dividing the number of falls by the total number of person-days for each time-interval. The rates are presented as falls per 1,000 person-days with 95 % confidence intervals. The rates represent the expected number of falls in 1,000 patients being at risk for 1 day in the respective time-interval.

The percentage of falls at day and night was calculated for the above defined time-intervals. Time for day and night was chosen according to the time intervals in which the majority of patients is either involved in daytime activities (6:00–20:59) or stay in bed for night’s rest (21:00–5:59).

To analyse if the influence of different covariates as risk factors for falls changes over time separate negative binomial regression models were applied for different time-intervals after admission. In order to reach a sufficient number of fall events for the regression models, the period of a complete week was used for each of the first 4 time intervals. The time period beyond 4 weeks (>28 days) was treated as 1 time interval. The multivariate models included gender, age, diagnosis, functional status (BI) and orientation. Cognition (DemTect) was not included in the model since it is not assessed directly at admission and is not reliable in disoriented patients. Falls are correlated with future falls, and multiple falls of 1 person either in 2 rehabilitation stays or within 1 rehabilitation stay are therefore not independent events. To account for this correlation of falls, a sensitivity analysis was performed. Only a patient’s first rehabilitation stay and only his/her first fall were included. For each time-period a Cox proportional hazard regression analysis was performed which included the same variables than the negative binomial regression models. Time after the first fall was censored.

## Results

The dataset consisted of 5,255 patients hospitalized in a geriatric rehabilitation clinic. 12 % of the patients had 2 hospitalizations and 2.4 % of the patients had more than 2 hospitalizations. The patients’ median age was 83.0 (interquartile range: 77.7; 87.3) years, the median length of stay was 22 (interquartile range: 20; 29) days. The most frequent reason for rehabilitation were femoral fractures (24.2 % of all patients) followed by stroke (9.7 %). One fourth of the patients was assessed as being disoriented at the time of admission and nearly one third was observed as having strong cognitive impairment, which was assessed by a cognitive test during the first week (DemTect 0–8 points). In total, 1,115 falls occurred within 109,457 person-days resulting in an overall fall rate of 10.2 falls/1,000 person-days. During the 5,255 rehabilitation stays, 560 (10.7 %) patients had 1 fall, 129 (2.5 %) patients 2 falls and 75 (1.4 %) patients more than 2 falls.

**Fig. 1:**
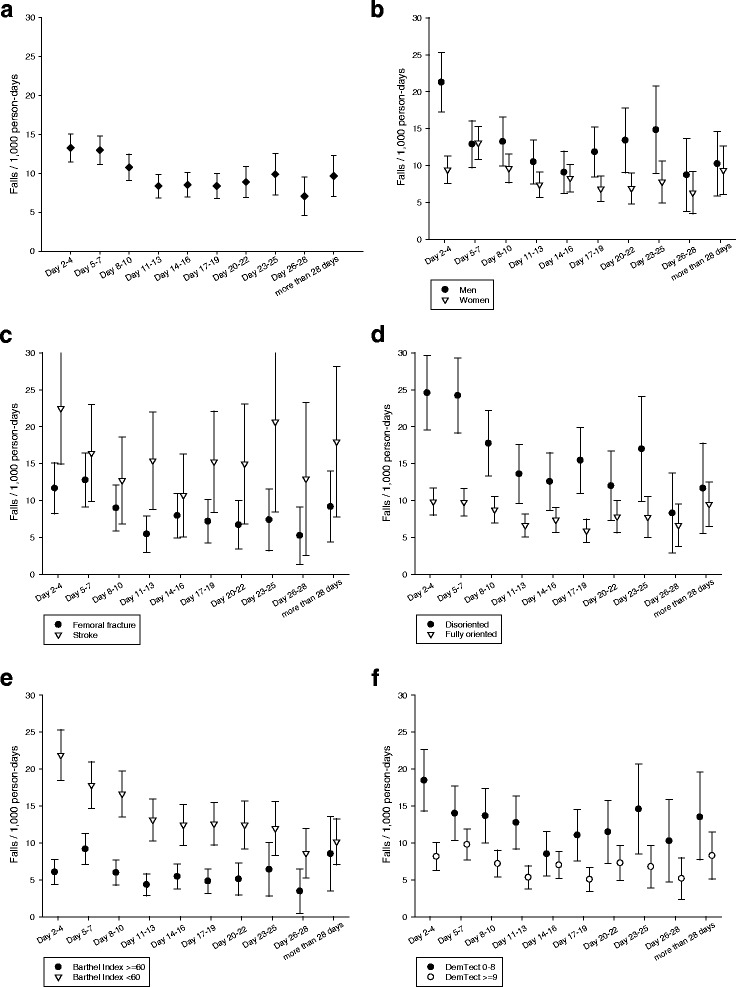
Fall rate as a function of time (**a**) after admission to the rehabilitation clinic, (**b**) stratified by gender, (**c**) by diagnosis, (**d**) by orientation, (**e**) by Barthel index, (**f**) by cognitive function (DemTect)

The fall risk was not constant during the length of stay in the rehabilitation clinic. In all patients combined, the fall rate was highest during the first week (13.3 falls/1,000 person-days) and decreased by about one third within the second and third week (Fig. 1a). This pattern was more pronounced in men than in women (Fig. 1b). For femoral fractures the pattern of fall risk over time was comparable to that of all patients combined. Patients with stroke had a generally higher fall risk than patients with femoral fracture but a less pronounced risk pattern over time (Fig. 1c). Clearly higher fall risks were observed during the first week in disoriented patients, in patients with a low functional status and in patients with strong cognitive impairment during the first week. Their fall risk dropped by nearly one half during the following weeks. In disoriented patients, for example, the fall rate decreased from 24.6 falls/1,000 person-days in day 2–4 to about 13 falls/1,000 person-days two weeks later. In contrast, no consistent time-dependent pattern was observed in fully oriented patients, in patients with a better functional status and in patients without or only slight cognitive impairment (Fig. 1d–f). The exact estimates are also presented in the Additional file 1: Tables A-G. Most of the above reported analyses showed an increase of the fall rate after the third week. This is mainly due to a selection of frail patients who got an extension of their stay beyond the usually granted rehabilitation period of 3 weeks. If this group of patients was analysed separately the observed increase of the fall rate after the third week disappeared (Additional file 1: Table B).

**Fig. 2:**
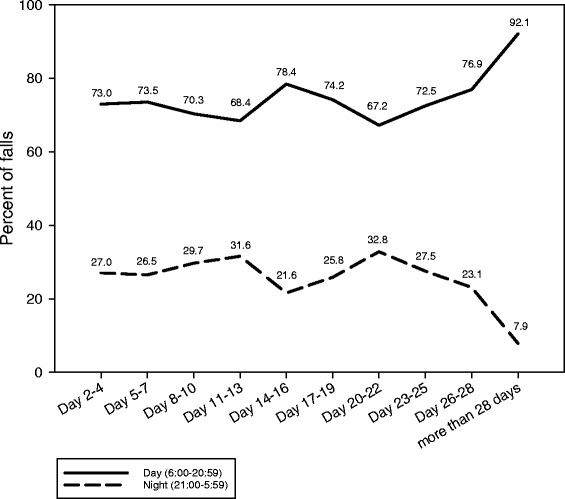
Distribution of falls between day and night stratified by time after admission to the rehabilitation clinic

Apart from the last time interval (>28 days), the distribution of falls between day and night was relatively constant over time (Fig. 2).

During the first week (day 2–7) the variables male gender, low functional status (Barthel-Index <60) and being disoriented increased the risk of falls significantly (Table 1). In the following weeks the relative risk for falls decreased considerably for being assessed as disoriented at admission and gradually for low functional status at admission. In contrast, the risk of falls for patients with the diagnosis stroke increased during the last two time intervals with incidence rate ratios of 2.06 (95 % confidence interval 1.13; 3.76) and 2.24 (95 % confidence interval 1.10; 4.58) (Table 1). The sensitivity analysis which considered only the first rehabilitation stay and the first fall did not make a meaningful change of the results (Additional file 1: Table H).

**Table 1:** Influence of gender, age, diagnosis and function at baseline on fall rate stratified by different time intervals after admission to the rehabilitation clinic

	Day 2–7	Day 8–14	Day 15–21	Day 22–28	>28 days
Person-days at risk (n)	30,666	33,219	28,565	11,641	5,366
Falls (n)	403	311	249	100	52
Incidence rate ratio (95 % confidence interval) ^b^					
Gender					
Women	1.00^a^	1.00^a^	1.00^a^	1.00^a^	1.00^a^
Men	1.45 (1.13;1.85)	1.17 (0.88;1.55)	1.49 (1.10;2.02)	1.61 (1.01;2.57)	0.97 (0.54;1.76)
Age (increase of one year)	1.00 (0.98;1.02)	1.00 (0.98;1.01)	1.01 (0.99;1.03)	0.97 (0.95;1.00)	0.97 (0.94;1.01)
Diagnosis					
All except femoral fracture and stroke	1.00^a^	1.00^a^	1.00^a^	1.00^a^	1.00^a^
Femoral fracture	0.80 (0.60;1.06)	0.64 (0.46;0.89)	0.75 (0.53;1.05)	0.93 (0.55;1.56)	1.18 (0.59;2.37)
Stroke	1.24 (0.88;1.74)	1.14 (0.78;1.66)	1.36 (0.89;2.07)	2.06 (1.13;3.76)	2.24 (1.10;4.58)
Barthel Index					
≥ 60	1.00^a^	1.00^a^	1.00^a^	1.00^a^	1.00^a^
< 60	2.22 (1.73;2.85)	2.42 (1.83;3.19)	2.30 (1.66;3.18)	1.78 (1.04;3.05)	1.07 (0.50;2.29)
Orientation					
Fully oriented	1.00^a^	1.00^a^	1.00^a^	1.00^a^	1.00^a^
Disoriented	1.99 (1.57;2.53)	1.66 (1.28;2.14)	1.54 (1.13;2.11)	1.41 (0.85;2.33)	1.18 (0.58;2.40)

^a^Reference group

^b^Negative binomial regression analysis; the columns represent independent models for each time interval

## Discussion

We found fall rates to be a function of time after admission to a geriatric rehabilitation clinic. A particularly pronounced risk pattern with high fall risks during the first days of sub-acute hospitalisation and decreasing risks thereafter were observed in subgroups of male patients, disoriented patients, patients with a low functional status or impaired cognition. Their fall risk within the first days was at least double as high as in the corresponding patients without these characteristics or limitations and decreased to nearly one half during the following weeks. As a consequence, the incidence rate ratio of baseline characteristics changed over time. In contrast, the distribution of falls between day and night seemed to be relatively constant during the rehabilitation period.

The observed mean fall rates were higher than in medical acute care settings [6, 7, 14] but in line with fall rates from acute and sub-acute geriatric units [4, 5, 15]. Rehabilitation is always a trade-off between risk-increasing mobilisation and safety. This may be one reason for the relatively high fall rates of patients treated in geriatric rehabilitation centres. Three studies mentioned already higher fall risks during the first days of a hospital stay [8–10]. These studies, however, have methodologic limitations and did not analyse our research question in detail.

The time-dependency of fall risk was particularly pronounced in disoriented people and in people with low functional status (BI <60). Explanations for the observed risk reduction over time in these two examples could be a gradually better orientation in the new environment, a declining delirium during rehabilitation or benefits due to rehabilitative measures particular in subgroups with a high risk of falls.

The identified risk factors of falls are identical to the results of a large body of literature [11, 16]. Our analyses add to the literature in the way that they demonstrate that the patient's clinical characteristics assessed at baseline may change over short time periods and require a continuous reappraisal of the patients’ fall risks. This is probably one of several reasons why fall risk assessment tools have only limited test properties [17].

Our data indicate that the fall risk was particularly a problem of patient subgroups during the early days after admission to the rehabilitation clinic. Therefore, interventions which focus on patients with disorientation or low physical function within the first days after admission appear to be appropriate. Generally, a close supervision of high-risk patients during the high-risk period seems to be reasonable. Another measure could be prompted voiding in order to reduce fall risk on the way to the toilet. Sensor mats which give an alarm if the patient gets out of bed may be an option particularly during night. Low-low beds could be used in agitated patients to make getting up more difficult and to reduce fall height. Hip protectors do not reduce fall risk but may reduce the risk of hip fractures [18]. Despite the obvious intuitive value of all the mentioned measures there is no direct empirical evidence for single measures in preventing falls or fall-related injuries in hospital [11]. Based on systematic reviews, the most appropriate approach to fall prevention in the hospital environment includes multifactorial interventions with multi-professional input [11, 19]. It is not clear if a risk reduction can be achieved already within the first days after admission. A large successful intervention study in sub-acute wards observed an effect even not before a stay of 45 days [20]. Two recently published studies from Australia evaluated the effect of multifactorial fall prevention measures in acute and sub-acute care. The first study applied exactly the above suggested measures in the acute hospital setting but did not reduce the rate of falls [21]. The other study was performed in aged care rehabilitation units and demonstrated a reduction of falls and fall-related injuries [22]. This study, however, used an individualised falls-prevention education programme for patients and may be therefore only of limited value in disoriented or cognitively impaired patients who have been shown in our study to be of particular risk during the first week. Our study does not tell which interventions will be finally successful. However, it shows clearly that future studies should focus on interventions which aim to influence the fall risk particularly in specific patient subgroups particularly during the high-risk period after admission.

Strengths of the study are the accurate documentation of falls and the exact consideration of time at risk in each analysed time-interval. The large number of patients and falls allowed evaluating fall rates even in small time periods. This study has also limitations to consider. First, only those falls were included in the analyses which were noticed by the staff. This results probably in an underestimation of the fall rate. Second, it cannot be completely excluded that the proportion of noticed and unnoticed falls changed during rehabilitation due to different time spent on individual care over time by the nurses. Third, the setting of a geriatric rehabilitation clinic is specific and its case mix and its daily routine differs from acute care settings, from organ-specific rehabilitative settings and even from geriatric settings of other countries. Therefore, the generalizability of the results has limitations.

## Conclusion

In summary, we found the fall risk during rehabilitation to be time-dependent. The strongest association between time and fall risk was observed in functionally limited patients with high risks during the first days after admission and declining risks thereafter. A substantial reduction of falls can be only expected if future fall prevention measures influence the fall risk in these subgroups particularly during the high-risk period after admission.

## Additional file

Additional file 1: Table A: Fall rate as a function of time after admission to the rehabilitation clinic (all patients combined). Table B: Fall rate as a function of time after admission to the rehabilitation clinic in patients with a length of stay of more than 22 days (all patients combined). Table C: Fall rate as a function of time after admission to the rehabilitation clinic stratified by gender. Table D: Fall rate as a function of time after admission to the rehabilitation clinic in patients with a femoral fracture or stroke. Table E: Fall rate as a function of time after admission to the rehabilitation clinic stratified by the degree of independence in the activities of daily living (Barthel Index). Table F: Fall rate as a function of time after admission to the rehabilitation clinic stratified by the degree of orientation. Table G: Fall rate as a function of time after admission to the rehabilitation clinic stratified by cognitive function (DemTect). Table H: Influence of gender, age, diagnosis and function at baseline on fall rate stratified by different time intervals after admission to the rehabilitation clinic (sensitivity analysis). (DOCX 38 kb)

## References

[1] Rapp K, Lamb SE, Klenk J, Kleiner A, Heinrich S, König H-H (2009). Fractures after nursing home admission: incidence and potential consequences. Osteoporos Int.

[2] Rapp K, Becker C, Lamb SE, Icks A, Klenk J (2008). Hip fractures in institutionalized elderly people: incidence rates and excess mortality. J Bone Miner Res Off J Am Soc Bone Miner Res.

[3] Rapp K, Cameron ID, Becker C, Kleiner A, Eckardt M, König H-H (2013). Femoral fracture rates after discharge from the hospital to the community. J Bone Miner Res Off J Am Soc Bone Miner Res.

[4] von Renteln-Kruse W, Krause T (2007). Incidence of in-hospital falls in geriatric patients before and after the introduction of an interdisciplinary team-based fall-prevention intervention. J Am Geriatr Soc.

[5] Schwendimann R, Bühler H, De Geest S, Milisen K (2008). Characteristics of hospital inpatient falls across clinical departments. Gerontology.

[6] Healey F, Scobie S, Oliver D, Pryce A, Thomson R, Glampson B (2008). Falls in English and Welsh hospitals: a national observational study based on retrospective analysis of 12 months of patient safety incident reports. Qual Saf Health Care.

[7] Bouldin ELD, Andresen EM, Dunton NE, Simon M, Waters TM, Liu M (2013). Falls among adult patients hospitalized in the United States: prevalence and trends. J Patient Saf.

[8] von Renteln-Kruse W, Krause T (2004). When do elderly in-hospital patients fall?. Age Ageing.

[9] Vassallo M, Sharma JC, Briggs RSJ, Allen SC (2003). Characteristics of early fallers on elderly patient rehabilitation wards. Age Ageing.

[10] Schwendimann R (1998). Frequency and circumstances of falls in acute care hospitals: a pilot study. Pflege.

[11] Oliver D, Healey F, Haines TP (2010). Preventing falls and fall-related injuries in hospitals. Clin Geriatr Med.

[12] Mahoney FI, Barthel DW (1965). Functional evaluation: the barthel index. Md State Med J.

[13] Kalbe E, Kessler J, Calabrese P, Smith R, Passmore AP, Brand M (2004). DemTect: a new, sensitive cognitive screening test to support the diagnosis of mild cognitive impairment and early dementia. Int J Geriatr Psychiatry.

[14] Heinze C, Halfens RJ, Dassen T (2007). Falls in German in-patients and residents over 65 years of age. J Clin Nurs.

[15] Nyberg L, Gustafson Y, Janson A, Sandman PO, Eriksson S (1997). Incidence of falls in three different types of geriatric care. A Swedish prospective study. Scand J Soc Med.

[16] Deandrea S, Lucenteforte E, Bravi F, Foschi R, La Vecchia C, Negri E (2010). Risk factors for falls in community-dwelling older people: a systematic review and meta-analysis. Epidemiol Camb Mass.

[17] Oliver D, Healy F (2009). Falls risk prediction tools for hospital inpatients: do they work?. Nurs Times.

[18] Parker MJ, Gillespie LD, Gillespie WJ (2000). Hip protectors for preventing hip fractures in the elderly. Cochrane Database Syst Rev.

[19] Cameron ID, Gillespie LD, Robertson MC, Murray GR, Hill KD, Cumming RG (2012). Interventions for preventing falls in older people in care facilities and hospitals. Cochrane Database Syst Rev.

[20] Haines TP, Bennell KL, Osborne RH, Hill KD (2004). Effectiveness of targeted falls prevention programme in subacute hospital setting: randomised controlled trial. BMJ.

[21] Barker AL, Morello RT, Wolfe R, Brand CA, Haines TP, Hill KD (2016). 6-PACK programme to decrease fall injuries in acute hospitals: cluster randomised controlled trial. BMJ.

[22] Hill A-M, McPhail SM, Waldron N, Etherton-Beer C, Ingram K, Flicker L (2015). Fall rates in hospital rehabilitation units after individualised patient and staff education programmes: a pragmatic, stepped-wedge, cluster-randomised controlled trial. Lancet Lond Engl.

