# Supplementary material for: Fall risk as a function of time after admission to sub-acute geriatric hospital units
Source: BMC Geriatr. 2016 Oct 7;16:173. doi: 10.1186/s12877-016-0346-7 (PMC5054540; doi:10.1186/s12877-016-0346-7)
Supplement: Additional file 1: — Table A: Fall rate as a function of time after admission to the rehabilitation clinic (all patients combined). Table B: Fall rate as a function of time after admission to the rehabilitation clinic in patients with a length of stay of more than 22 days (all patients combined). Table C: Fall rate as a function of time after admission to the rehabilitation clinic stratified by gender. Table D: Fall rate as a function of time after admission to the rehabilitation clinic in patients with a femoral fracture or stroke. Table E: Fall rate as a function of time after admission to the rehabilitation clinic stratified by the degree of independence in the activities of daily living (Barthel Index). Table F: Fall rate as a function of time after admission to the rehabilitation clinic stratified by the degree of orientation. Table G: Fall rate as a function of time after admission to the rehabilitation clinic stratified by cognitive function (DemTect). Table H: Influence of gender, age, diagnosis and function at baseline on fall rate stratified by different time intervals after admission to the rehabilitation clinic (sensitivity analysis). (DOCX 38 kb) [file 12877_2016_346_MOESM1_ESM.docx]

SUPPLEMENTARY FILES

Supplement Table A: Fall rate as a function of time after admission to the rehabilitation clinic (all patients combined)

|  | **All patients** | | |
| --- | --- | --- | --- |
| Time interval after admission (days) | pd* (n) | Falls (n) | Falls/1000 pd  (95% CI)^†^ |
| 2-4 | 15584 | 207 | 13.28 (11.47; 15.09) |
| 5-7 | 15082 | 196 | 13.00 (11.18; 14.82) |
| 8-10 | 14564 | 157 | 10.78 (9.09; 12.47) |
| 11-13 | 14069 | 118 | 8.39 (6.87; 9.90) |
| 14-16 | 13568 | 116 | 8.55 (6.99; 10.11) |
| 17-19 | 12731 | 107 | 8.40 (6.81; 10.00) |
| 20-22 | 8766 | 78 | 8.90 (6.92; 10.87) |
| 23-25 | 5346 | 53 | 9.91 (7.24; 12.58) |
| 26-28 | 4381 | 31 | 7.08 (4.59; 9.57) |
| >28 | 5366 | 52 | 9.69 (7.06; 12.32) |

* pd: person-days

^†^ Falls/1000 person-days (95% confidence interval)

Supplement Table B: Fall rate as a function of time after admission to the rehabilitation clinic in patients with a length of stay of more than 22 days (all patients combined)

|  | **All patients** | | |
| --- | --- | --- | --- |
| Time interval after admission (days) | pd* (n) | Falls (n) | Falls/1000 pd  (95% CI)^†^ |
| 2-4 | 5742 | 85 | 14.80 (11.66; 17.95) |
| 5-7 | 5742 | 74 | 12.89 (9.95; 15.82) |
| 8-10 | 5742 | 71 | 12.37 (9.49; 15.24) |
| 11-13 | 5742 | 57 | 9.93 (7.35; 12.50) |
| 14-16 | 5742 | 57 | 9.93 (7.35; 12.50) |
| 17-19 | 5742 | 69 | 12.02 (9.18; 14.85) |
| 20-22 | 5742 | 59 | 10.28 (7.65; 12.90) |
| 23-25 | 5346 | 53 | 9.91 (7.24; 12.58) |
| 26-28 | 4381 | 31 | 7.08 (4.59; 9.57) |
| >28 | 5366 | 52 | 9.69 (7.06; 12.32) |

* pd: person-days

^†^ Falls/1000 person-days (95% confidence interval)

Supplement Table C: Fall rate as a function of time after admission to the rehabilitation clinic stratified by gender

|  | **Women** | | |  | **Men** | | |
| --- | --- | --- | --- | --- | --- | --- | --- |
| Time interval after admission (days) | pd* (n) | Falls (n) | Falls/1000 pd  (95% CI)^†^ |  | pd* (n) | Falls (n) | Falls/1000 pd  (95% CI)^†^ |
| 2-4 | 10507 | 99 | 9.42 (7.57; 11.28) |  | 5077 | 108 | 21.27 (17.26; 25.28) |
| 5-7 | 10191 | 133 | 13.05 (10.83; 15.27) |  | 4891 | 63 | 12.88 (9.70; 16.06) |
| 8-10 | 9882 | 95 | 9.61 (7.68; 11.55) |  | 4682 | 62 | 13.24 (9.95; 16.54) |
| 11-13 | 9587 | 71 | 7.41 (5.68; 9.13) |  | 4482 | 47 | 10.49 (7.49; 13.48) |
| 14-16 | 9272 | 77 | 8.30 (6.45; 10.16) |  | 4296 | 39 | 9.08 (6.23; 11.93) |
| 17-19 | 8760 | 60 | 6.85 (5.12; 8.58) |  | 3971 | 47 | 11.84 (8.45; 15.22) |
| 20-22 | 6083 | 42 | 6.90 (4.82; 8.99) |  | 2683 | 36 | 13.42 (9.03; 17.80) |
| 23-25 | 3728 | 29 | 7.78 (4.95; 10.61) |  | 1618 | 24 | 14.83 (8.90; 20.77) |
| 26-28 | 3005 | 19 | 6.32 (3.48; 9.17) |  | 1376 | 12 | 8.72 (3.79; 13.66) |
| >28 | 3315 | 31 | 9.35 (6.06; 12.64) |  | 2051 | 21 | 10.24 (5.86; 14.62) |

* pd: person-days

^†^ Falls/1000 person-days (95% confidence interval)

Supplement Table D: Fall rate as a function of time after admission to the rehabilitation clinic in patients with a femoral fracture or stroke

|  | **Femoral fracture** | | |  | **Stroke** | | |
| --- | --- | --- | --- | --- | --- | --- | --- |
| Time interval after admission (days) | pd* (n) | Falls (n) | Falls/1000 pd  (95% CI)^†^ |  | pd* (n) | Falls (n) | Falls/1000 pd  (95% CI)^†^ |
| 2-4 | 3772 | 44 | 11.66 (8.22; 15.11) |  | 1513 | 34 | 22.47 (14.92; 30.03) |
| 5-7 | 3680 | 47 | 12.77 (9.12; 16.42) |  | 1462 | 24 | 16.42 (9.85; 22.98) |
| 8-10 | 3563 | 32 | 8.98 (5.87; 12.09) |  | 1416 | 18 | 12.71 (6.84; 18.58) |
| 11-13 | 3481 | 19 | 5.46 (3.00; 7.91) |  | 1363 | 21 | 15.41 (8.82; 22.00) |
| 14-16 | 3395 | 27 | 7.95 (4.95; 10.95) |  | 1309 | 14 | 10.70 (5.09; 16.30) |
| 17-19 | 3204 | 23 | 7.18 (4.24; 10.11) |  | 1247 | 19 | 15.24 (8.39; 22.09) |
| 20-22 | 2384 | 16 | 6.71 (3.42; 10.00) |  | 869 | 13 | 14.96 (6.83; 23.09) |
| 23-25 | 1621 | 12 | 7.40 (3.21; 11.59) |  | 533 | 11 | 20.64 (8.44; 32.83) |
| 26-28 | 1332 | 7 | 5.26 (1.36; 9.15) |  | 464 | 6 | 12.93 (2.58; 23.28) |
| >28 | 1526 | 14 | 9.17 (4.37; 13.98) |  | 668 | 12 | 17.96 (7.80; 28.13) |

* pd: person-days

^†^ Falls/1000 person-days (95% confidence interval)

Supplement Table E: Fall rate as a function of time after admission to the rehabilitation clinic stratified by the degree of independence in the activities of daily living (Barthel Index)

|  | **Barthel Index ≥60** | | |  | **Barthel Index <60** | | |
| --- | --- | --- | --- | --- | --- | --- | --- |
| Time interval after admission (days) | pd* (n) | Falls (n) | Falls/1000 pd  (95% CI)^†^ |  | pd* (n) | Falls (n) | Falls/1000 pd  (95% CI)^†^ |
| 2-4 | 8261 | 50 | 6.05 (4.37; 7.73) |  | 7129 | 156 | 21.88 (18.45; 25.32) |
| 5-7 | 8070 | 74 | 9.17 (7.08; 11.26) |  | 6845 | 122 | 17.82 (14.66; 20.99) |
| 8-10 | 7856 | 47 | 5.98 (4.27; 7.69) |  | 6560 | 109 | 16.62 (13.50; 19.74) |
| 11-13 | 7606 | 33 | 4.34 (2.86; 5.82) |  | 6338 | 83 | 13.10 (10.28; 15.91) |
| 14-16 | 7346 | 40 | 5.45 (3.76; 7.13) |  | 6117 | 76 | 12.42 (9.63; 15.22) |
| 17-19 | 6841 | 33 | 4.82 (3.18; 6.47) |  | 5807 | 73 | 12.57 (9.69;15.45) |
| 20-22 | 4118 | 21 | 5.10 (2.92; 7.28) |  | 4588 | 57 | 12.42 (9.20;15.65) |
| 23-25 | 1871 | 12 | 6.41 (2.78; 10.04) |  | 3432 | 41 | 11.95 (8.29;15.60) |
| 26-28 | 1445 | 5 | 3.46 (0.43; 6.49) |  | 2908 | 25 | 8.60 (5.23;11.97) |
| >28 | 1289 | 11 | 8.53 (3.49; 13.58) |  | 4036 | 41 | 10.16 (7.05;13.27) |

* pd: person-days (does not sum up to 100% due to missings in the variable ‘Barthel Index’)

^†^ Falls/1000 person-days (95% confidence interval)

Supplement Table F: Fall rate as a function of time after admission to the rehabilitation clinic stratified by the degree of orientation

|  | **Fully oriented** | | |  | **Disoriented** | | |
| --- | --- | --- | --- | --- | --- | --- | --- |
| Time interval after admission (days) | pd* (n) | Falls (n) | Falls/1000 pd  (95% CI)^†^ |  | pd* (n) | Falls (n) | Falls/1000 pd  (95% CI)^†^ |
| 2-4 | 11268 | 111 | 9.85 (8.02:11.68) |  | 3742 | 92 | 24.59 (19.56;29.61) |
| 5-7 | 10942 | 107 | 9.78 (7.93;11.63) |  | 3594 | 87 | 24.21 (19.12;29.29) |
| 8-10 | 10601 | 93 | 8.77 (6.99;10.56) |  | 3436 | 61 | 17.75 (13.30;22.21) |
| 11-13 | 10261 | 68 | 6.63 (5.05;8.20) |  | 3309 | 45 | 13.60 (9.63;17.57) |
| 14-16 | 9913 | 73 | 7.36 (5.67;9.05) |  | 3181 | 40 | 12.57 (8.68;16.47) |
| 17-19 | 9316 | 55 | 5.90 (4.34;7.46) |  | 2979 | 46 | 15.44 (10.98;19.90) |
| 20-22 | 6391 | 50 | 7.82 (5.65;9.99) |  | 2085 | 25 | 11.99 (7.29;16.69) |
| 23-25 | 3872 | 30 | 7.75 (4.98;10.52) |  | 1295 | 22 | 16.99 (9.89;24.09) |
| 26-28 | 3156 | 21 | 6.65 (3.81;9.50) |  | 1085 | 9 | 8.29 (2.88; 13.71) |
| >28 | 3997 | 38 | 9.51 (6.48;12.53) |  | 1201 | 14 | 11.66 (5.55;17.76) |

* pd: person-days (does not sum up to 100% due to missings in the variable ‘orientation’)

^†^ Falls/1000 person-days (95% confidence interval)

Supplement Table G: Fall rate as a function of time after admission to the rehabilitation clinic stratified by cognitive function (DemTect)

|  | **DemTect ≥9** | | |  | **DemTect 0-8** | | |
| --- | --- | --- | --- | --- | --- | --- | --- |
| Time interval after admission (days) | pd* (n) | Falls (n) | Falls/1000 pd  (95% CI)^†^ |  | pd* (n) | Falls (n) | Falls/1000 pd  (95% CI)^†^ |
| 2-4 | 8838 | 72 | 8.15 (6.26; 10.03) |  | 4061 | 75 | 18.47 (14.29; 22.65) |
| 5-7 | 8687 | 85 | 9.78 (7.70; 11.86) |  | 3997 | 56 | 14.01 (10.34; 17.68) |
| 8-10 | 8483 | 61 | 7.19 (5.39; 9.00) |  | 3879 | 53 | 13.66 (9.98; 17.34) |
| 11-13 | 8260 | 44 | 5.33 (3.75; 6.90) |  | 3768 | 48 | 12.74 (9.14; 16.34) |
| 14-16 | 8004 | 56 | 7.00 (5.16; 8.83) |  | 3642 | 31 | 8.51 (5.52; 11.51) |
| 17-19 | 7508 | 38 | 5.06 (3.45;6.67) |  | 3443 | 38 | 11.04 (7.53; 14.55) |
| 20-22 | 5081 | 37 | 7.28 (4.94;9.63) |  | 2436 | 28 | 11.49 (7.24; 15.75) |
| 23-25 | 3099 | 21 | 6.78 (3.88;9.67) |  | 1510 | 22 | 14.57 (8.48; 20.66) |
| 26-28 | 2510 | 13 | 5.18 (2.36;7.99) |  | 1266 | 13 | 10.27 (4.69; 15.85) |
| >28 | 3143 | 26 | 8.27 (5.09;11.45) |  | 1407 | 19 | 13.50 (7.43; 19.58) |

* pd: person-days

^†^ Falls/1000 person-days (95% confidence interval)

Supplement Table H: Influence of gender, age, diagnosis and function at baseline on fall rate stratified by different time intervals after admission to the rehabilitation clinic **(sensitivity analysis)**

|  | **Day 2-7** | **Day 8-14** | **Day 15-21** | **Day 22-28** | **> 28 days** |
| --- | --- | --- | --- | --- | --- |
| Falls (n) | 278 | 175 | 128 | 46 | 17 |
|  | **Hazard ratio (95% confidence interval) ^†^** | | | | |
| Gender |  |  |  |  |  |
| Women | 1.00* | 1.00* | 1.00* | 1.00* | 1.00* |
| Men | 1.30 (1.01;1.67) | 1.15 (0.83;1.57) | 1.40 (0.96;2.03) | 1.38 (0.74;2.57) | 1.58 (0.56;4.47) |
| Age (increase of one year) | 1.00 (0.98;1.01) | 0.99 (0.97;1.01) | 1.02 (1.00;1.05) | 0.99 (0.94;1.03) | 1.05 (0.97;1.13) |
| Diagnosis |  |  |  |  |  |
| All except femoral fracture  and stroke | 1.00* | 1.00* | 1.00* | 1.00* | 1.00* |
| Femoral fracture | 0.81 (0.60;1.10) | 0.58 (0.38;0.86) | 0.82 (0.53;1.27) | 1.06 (0.53;2.15) | 1.55 (0.48;5.02) |
| Stroke | 1.31 (0.93;1.84) | 0.93 (0.58;1.49) | 1.46 (0.89;2.39) | 2.36 (1.10;5.07) | 2.69 (0.74;9.78) |
| Barthel Index |  |  |  |  |  |
| ≥60 | 1.00* | 1.00* | 1.00* | 1.00* | 1.00* |
| <60 | 2.40 (1.84;3.12) | 2.27 (1.64;3.13) | 2.71 (1.84;3.98) | 1.30 (0.67;2.52) | 2.46 (0.55;11.06) |
| Orientation |  |  |  |  |  |
| Fully oriented | 1.00* | 1.00* | 1.00* | 1.00* | 1.00* |
| Disoriented | 1.76 (1.37;2.26) | 1.71 (1.24;2.35) | 1.07 (0.72;1.59) | 1.18 (0.61;2.26) | 0.44 (0.10;1.99) |

* Reference group

^†^ Cox proportional regression; the columns represent independent models for each time interval
